# The antiviral activity of kaempferol against pseudorabies virus in mice

**DOI:** 10.1186/s12917-021-02953-3

**Published:** 2021-07-18

**Authors:** Lixia Li, Rui Wang, Huaiyue Hu, Xu Chen, Zhongqiong Yin, Xiaoxia Liang, Changliang He, Lizi Yin, Gang Ye, Yuanfeng Zou, Guizhou Yue, Huaqiao Tang, Renyong Jia, Xu Song

**Affiliations:** 1 Natural Medicine Research Center, College of Veterinary Medicine, Sichuan Agricultural University, 611130 Chengdu, China; 2 College of Science, Sichuan Agricultural University, 625014 Ya’an, China

**Keywords:** Kaempferol, Pseudorabies virus, Antiviral activity

## Abstract

**Background:**

Pseudorabies virus (PRV), a member of the *Alphaherpesviruses*, is one of the most important pathogens that harm the global pig industry. Accumulated evidence indicated that PRV could infect humans under certain circumstances, inducing severe clinical symptoms such as acute human encephalitis. Currently, there are no antiviral drugs to treat PRV infections, and vaccines available only for swine could not provide full protection. Thus, new control measures are urgently needed.

**Results:**

In the present study, kaempferol exhibited anti-PRV activity in mice through improving survival rate by 22.22 %, which was higher than acyclovir (Positive control) with the survival rate of 16.67 % at 6 days post infection (dpi); meanwhile, the survival rate was 0 % at 6 dpi in the infected-untreated group. Kaempferol could inhibit the virus replication in the brain, lung, kidney, heart and spleen, especially the viral gene copies were reduced by over 700-fold in the brain, which was further confirmed by immunohistochemical examination. The pathogenic changes induced by PRV infection in these organs were also alleviated. The transcription of the only immediate-early gene IE180 in the brain was significantly inhibited by kaempferol, leading to the decreased transcriptional levels of the early genes (EPO and TK). The expression of latency-associated transcript (LAT) was also inhibited in the brain, which suggested that kaempferol could inhibit PRV latency. Kaempferol-treatment could induce higher levels of IL-1β, IL-4, IL-6, TNF-α and IFN-γ in the serum at 3 dpi which were then declined to normal levels at 5 dpi.

**Conclusions:**

These results suggested that kaempferol was expected to be a new alternative control measure for PRV infection.

## Background

Pseudorabies virus (PRV), a member of the *Alphaherpesviruses*, is the causative agent of pseudorabies which was also called Aujeszky’s disease (AD) [1]. Apart from the direct effects of AD, the trading of PRV-infected pigs and their products are globally restricted. Most mammals are susceptible to PRV, and infections often lead to death in ruminants, carnivores and rodents except pigs which are the only natural hosts for PRV [2]. Whether PRV is able to infect humans is still controversial. In 1914, suspected human infections with PRV were first reported, then 8 human cases of infection with PRV (22 humans) were reported over the past 100 years [3]. Recently, four cases of acute human encephalitis caused by PRV variant strain were reported and a PRV strain was firstly isolated from these patients [4]. Thus, there is increasing evidence that PRV could infect humans under certain circumstances. In addition to concern about the control of PRV infection, PRV has been used as a model organism to study the molecular biology of herpesviruses [5]. The PRV-infected pigs exhibit a variety of clinical symptoms, including high mortality in piglets, growth retardation in adult pigs, and reproductive failure in sows, which causes substantial economic losses to swine industry [6]. PRV usually induces latent-infection in adult pigs, and the hosts behave normally and show no clinical symptoms [7]. However, once the host is stressed or its resistance becomes reduced, PRV can be re-activated and a large number of virions were produced to infect central nervous system [8], causing re-emergence of PRV infection, even the host becomes a source of infection [9]. The PRV infections are mainly achieved by controlling transcription levels. The gene expression of PRV can be divided into three stages: immediate early gene (IE), early gene (E) and late gene (L). In those stages, IE180 is the only immediate early gene and its expression product is an effective transcriptional activator of viral genes involved in DNA replication and RNA transcription [10]. The early genes that have been reported are EP0, TK and UL54 [11–13]. EP0 is expressed as an early protein in PRV replication cycle, which can transactivate viral promoters, such as IE180, TK and gG [14]. The viral gene is not expressed during latent infection, and only a segment of the non-coding region of PRV genome transcribes the latency-associated transcript (LAT) [15].

Although AD has been eradicated from domestic pig populations in the United States and some European countries, PRV epidemic is especially prominent in regions of South America and Asia [16]. At present, there are no drugs available for PRV infections, and vaccination is still the main measure to prevent AD. Many vaccines against PRV have been successfully developed, including inactivated and attenuated vaccines and genetically engineered vaccines [17, 18]. Although vaccines can effectively control PRV spread, it doesn’t protect against latency of wild-type virus and PRV excretion [19]. Since 2011, the outbreaks of PRV variants in Bartha-K61 vaccinated pigs have been constantly reported, and the origin of these variant viruses remains to be addressed [20]. The variability of viral strains and the insecurity of attenuated vaccines are a serious threat to the pig industry. Thus, newly control measures are urgently needed.

Kaempferol, 3, 5, 7 - trihydroxy − 2 - (4-hydroxyphenyl) − 4* H* − 1 - benzopyran − 4 - one, is a natural flavonol which is mainly derived from the rhizome of the ginger family and present in different plant species, such as tea, broccoli, purple cabbage, beans, chicory, leeks, tomatoes, strawberries and grapes [21, 22]. It is a tetrahydroxy flavone in which the four hydroxy groups are located at positions 3, 5, 7, and 4′ [23]. It has received widespread attention because of its anticancer, anti-inflammatory, antioxidant, antibacterial and antiviral effects as well as treatment of diabetes and osteoporosis [3, 24–26]. Previously, we found that kaempferol possesses the ability to inhibit PRV replication in a dose-dependent manner *in vitro* with a 50 % inhibitory concentration of 25.57 µM (Unpublished data). In order to development of a complementary control measure for PRV infections, the present study evaluated the anti-PRV potency of kaempferol in PRV-infected mice.

## Results

### Survival rate

**Fig. 1:**
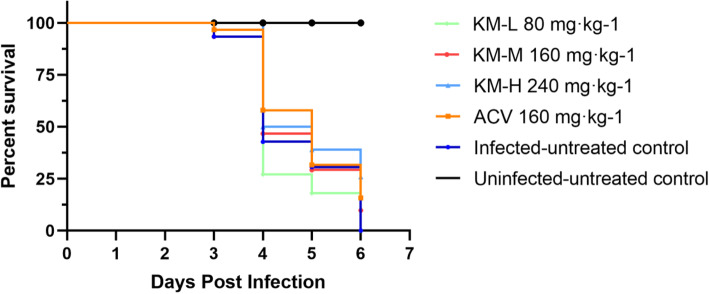
The survival rate of mice in each group depicted by Kaplan-Meier survival plots. The survival rate of each group was calculated according to the formula: survival rate = number of surviving mice / total number of mice. The total number of mice on days 4, 5 and 6 were the number after removal of 4 mice on days 3, 4 and 5, respectively. The KM-H, KM-M and KM-L represent the PRV-infected groups which were treated with kaempferol at doses of 240, 160 and 80 mg/kg• body weight, respectively. ACV, the acyclovir-treated group (160 mg/kg• body weight)

After artificial infection by PRV for 2 days, symptoms similar to natural infections appeared. The mice began to show itching symptoms. The survival rate of mice in each group was shown in Fig. 1. There were no deaths in each group at 2 days post infection (dpi). At 3 dpi, except for the uninfected-untreated and KM-H groups, the infected mice in other groups began to die with neurological symptoms such as severe and uncontrollable pruritus (itchiness). The acute encephalitis induced by host immune response and injury in the peripheral nervous system were the major cause of the death [27]. The survival rates of the KM-L, KM-M and infected-untreated groups were below 50 % at 4 dpi; in the KM-H and acyclovir groups, the survival rates were 50 and 57.69 %, respectively. The KM-H group exhibited the highest survival rate (36.36 %) at 5 dpi, while the survival rate of the acyclovir group was 31.81 %. At 6 dpi, all mice in the KM-L and infected-untreated groups died. The survival rate of the KM-H group was 22.22 %, which was higher than acyclovir group with survival rate of 16.67 %. These results suggested that kaempferol at the dose of 240 mg/kg had higher activity to protect mice from PRV- induced death than acyclovir did.

### Organ coefficient

**Fig. 2:**
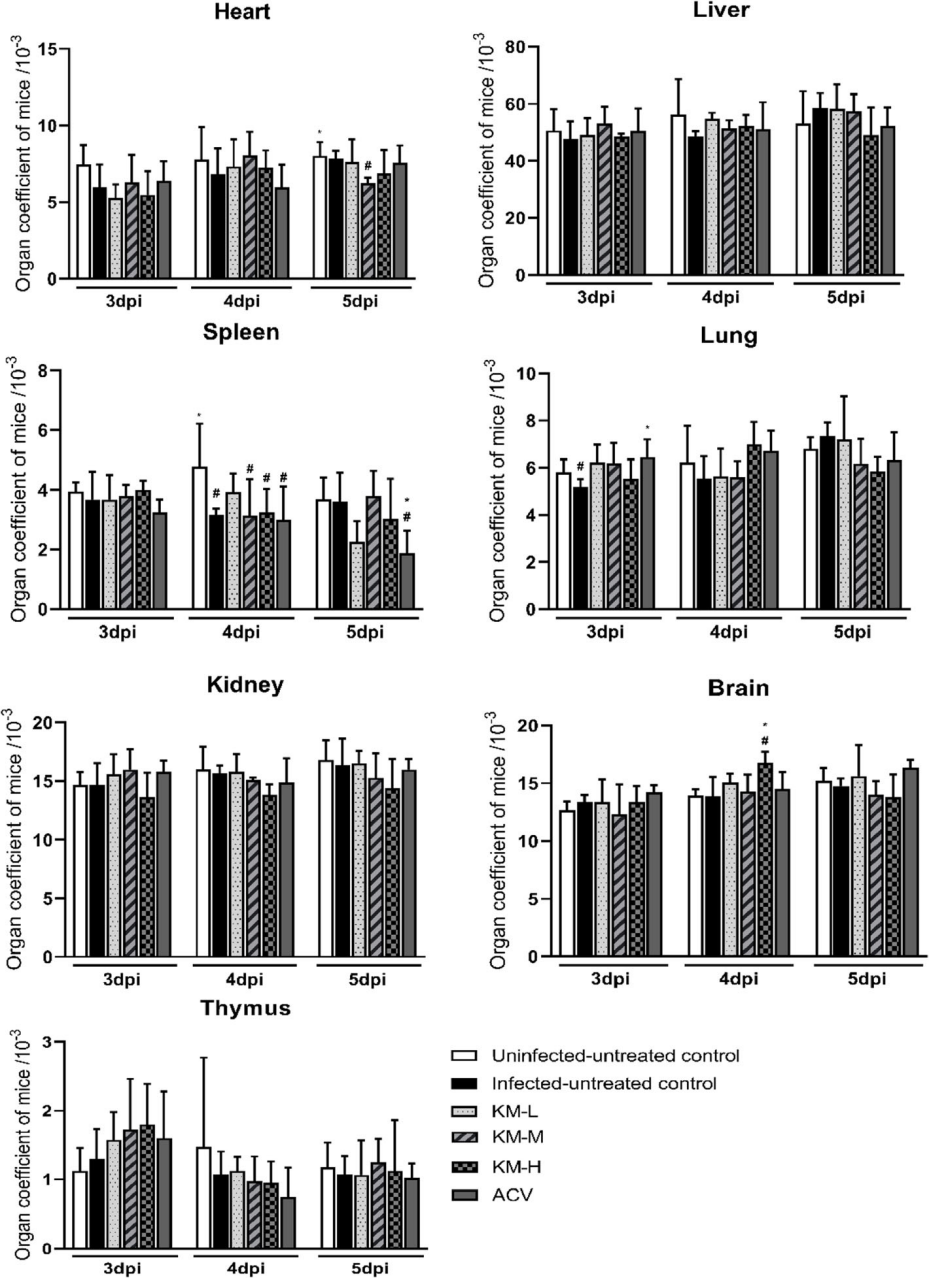
Organ coefficient of mice. The heart, liver, spleen, lung, kidney, thymus and brain were collected and weighed at 3,4 and 5 dpi (*n* = 4). The relative organ weight was calculated according to the formula: organ coefficient (mg / g) = organ weight / body weight. “*” represents the significant differences (*p* < 0.05) observed between the infected-untreated group and infected-treated groups, and “#” represents the significant differences (*p* < 0.05) observed between the uninfected-untreated group and infected groups

Organ coefficient, the ratio of organ weight to body weight, is a commonly used index in toxicology test. The ratio of each organ to body weight is relatively constant under normal conditions. However, the organ coefficient would alter with the change of damaged organ weight after animals are poisoned [28]. The result of organ coefficient was shown in Fig. 2. PRV infection significantly decreased the lung coefficient at 3 dpi, which was significantly increased by acyclovir-treatment. At 4 dpi, PRV infection significantly decreased the spleen coefficient, and the brain coefficient of KM-H group was significantly increased. Acyclovir-treatment significantly decreased the spleen coefficient at 5dpi.

### Virus load

**Fig. 3:**
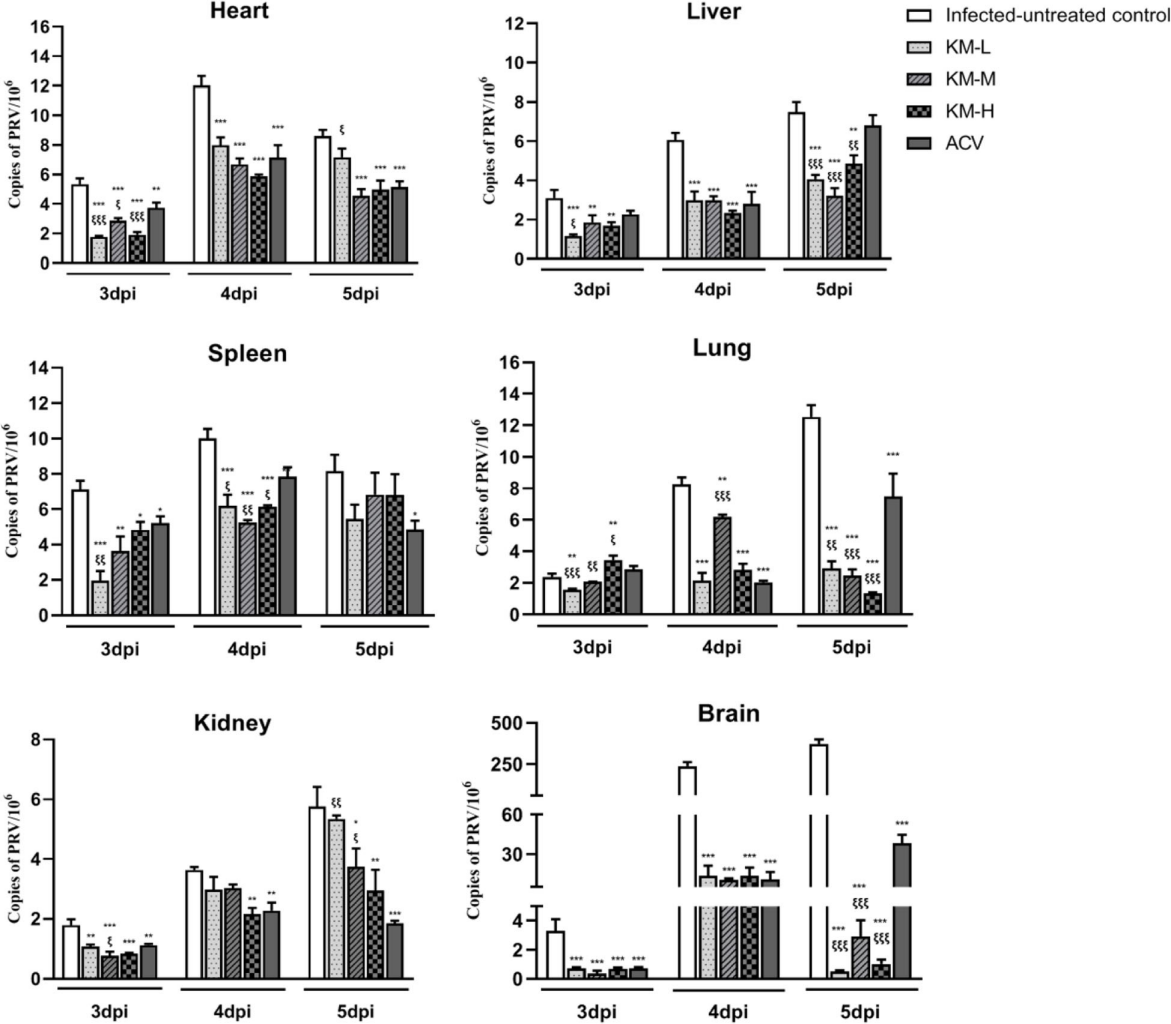
Virus load of heart, liver, spleen, lung, kidney and brain in each group (*n* = 4). The mice were randomly dissected at 3, 4, and 5 dpi to collect these tissues. After homogenization with liquid nitrogen, the tissue samples (25 mg per sample) were used for DNA extraction. The virus gene copies were detected by FQ-PCR. The KM-H, KM-M and KM-L represent the PRV-infected groups which were treated with kaempferol at doses of 240, 160 and 80 mg/kg• body weight, respectively. ACV, the acyclovir-treated group (160 mg/kg• body weight). Symbols “*, ** and ***” represent *p* < 0.05, *p* < 0.01 and *p* < 0.001, respectively, between the infected-untreated control and the treated groups. Symbols “ξ, ξξ and ξξξ” represent *p* < 0.05, *p* < 0.01 and *p* < 0.001, respectively, between the acyclovir group and kaempferol groups

The virus load in heart, spleen, liver, lung, kidney and brain were reflected by viral gene copies which were determined through FQ-PCR. As can be seen from Fig. 3, with the increase of infection time, the viral loads of the infected-untreated group showed an upward trend in liver, lung and brain. The viral loads reached the peak at 4 dpi in heart, spleen and kidney. After treated with acyclovir and kaempferol, the viral loads were significantly reduced in the test organs. In liver, lung and brain, the viral loads of kaempferol-treated groups were significantly lower than that of acyclovir-treated group, and kaempferol at a dose of 240 mg/kg exhibited the highest potency.

**Fig. 4:**
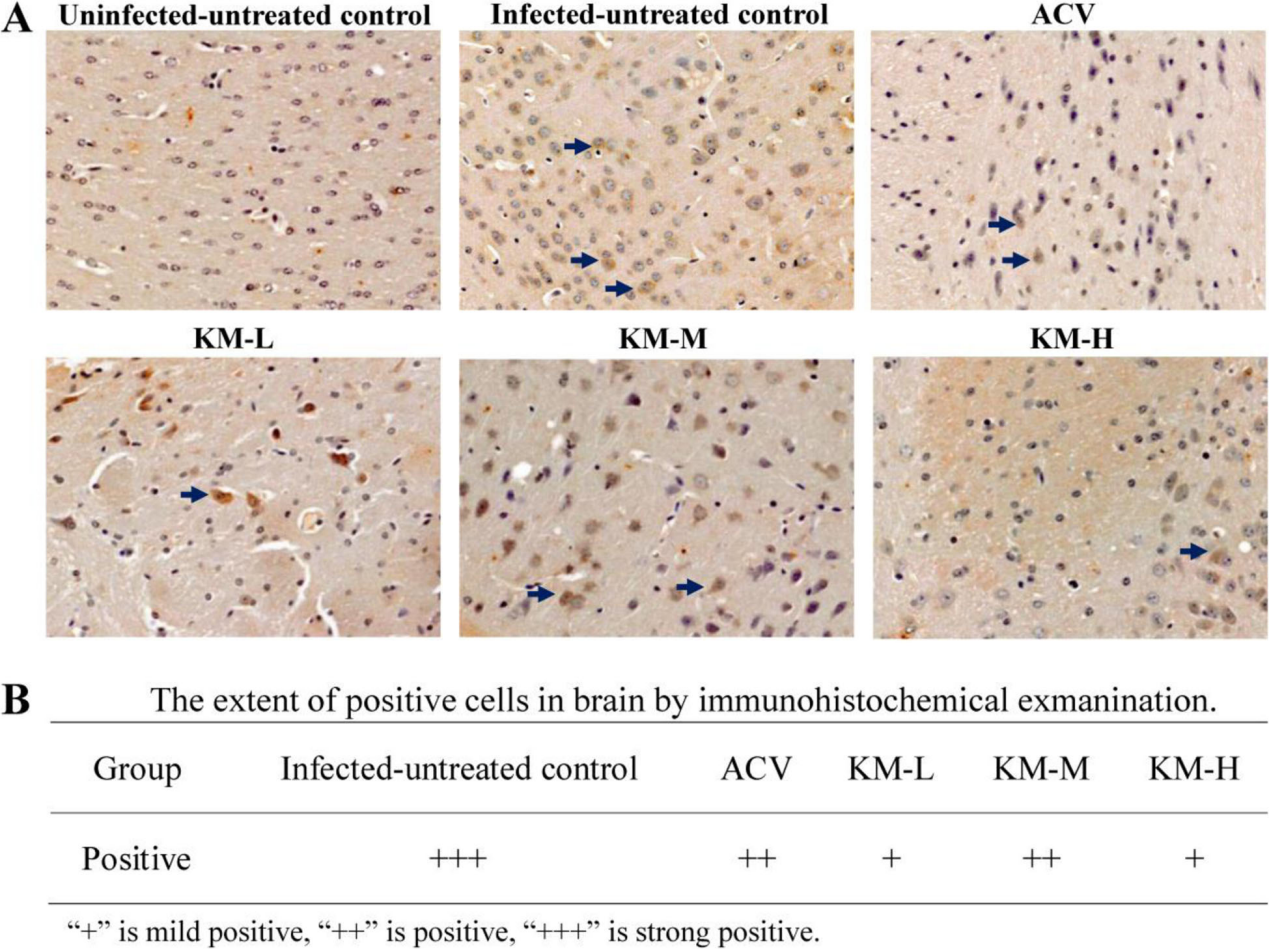
Immunohistochemical examination of the brain at 5 dpi. The KM-H, KM-M and KM-L represent the PRV-infected groups which were treated with kaempferol at doses of 240, 160 and 80 mg/kg• body weight, respectively. ACV, the acyclovir-treated group (160 mg/kg• body weight). **A** DAB chromogen substrates were used for antigen visualization. The SABC staining positive cells mainly showed brownish-yellow (indicated by arrows), and the section Background mainly showed light yellow. Each tissue undergoes three SABC stains to check its repeatability. The uninfected–untreated control had no obvious positive cells, and the infected-untreated group had the most number of positive cells. Compared with the infected-untreated group, the number of positive cells in the treated group decreased. **B** No brownish-yellow cells detected in the uninfected-untreated control were negative; the scattered distribution of brownish-yellow cells in the tissue is less than 5 % indicates mildly positive; the proportion of brownish-yellow cells in the tissue is 5 − 50 % indicates positive; the proportion of brownish-yellow cells in the tissue is greater than 50 % indicates strong positive

As the gene copies of PRV in the brain were the highest, immunohistochemical examination was employed for further detection of virus reproduction in the brain. The brain tissue slides were stained by SABC method, and the positive cells mainly showed brown and the section Background mainly showed light yellow. As shown in Fig. 4, there were no obvious positive cells in the uninfected-untreated group, and the number of positive cells in the infected-untreated group was higher than those of the treated groups, which showed the consistent results with the viral load study.

### Histopathological examination

Viral infections usually cause damages to healthy tissues, thus the heart, liver, spleen, lung, kidney and brain in each group at 5dpi were further subjected to histopathological examination. The following pathological changes were found.

**Fig. 5:**
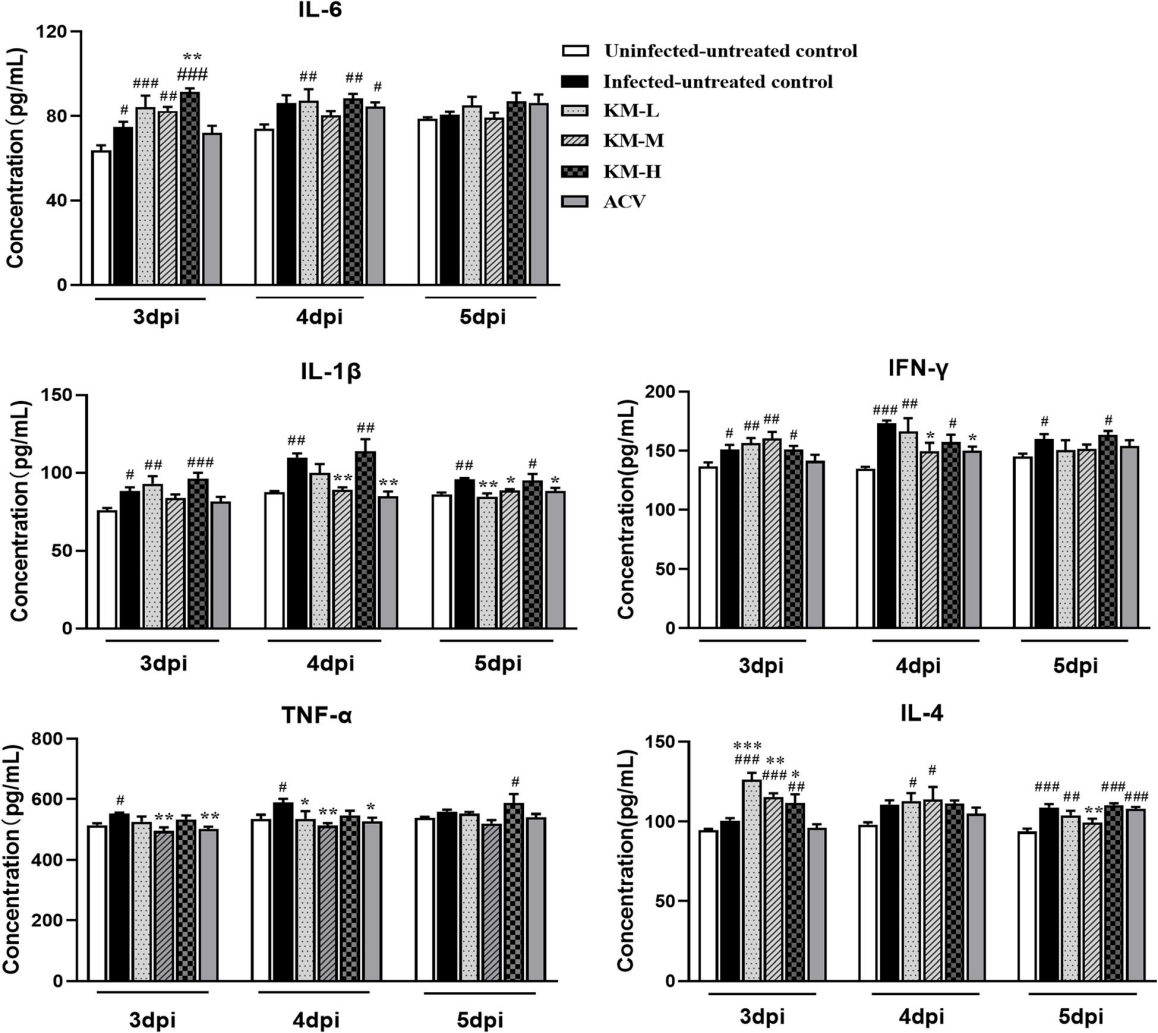
Histopathological examination of the tissues including heart, liver and spleen on 5dpi. In the heart (**A-F**), PRV infection causes breakage of myocardial fibers and disordered arrangement (denoted by arrowhead “←”), the blood vessel wall of the heart appears shedding to varying degrees (denoted by arrowhead “→”), and localized vacuolar degeneration (denoted by arrowhead “↑”). In the liver (**G-L**), the main histopathological changes are shedding of blood vessel walls (denoted by arrowhead “←”), disordered arrangement of hepatic cords (denoted by arrowhead “→”), hepatocellular swelling (denoted by arrowhead “↓”), and foam-like changes (denoted by arrowhead “↑”). In the spleen (**M-R**), the lesions showed a decrease in the area of the red pulp area (denoted by arrowhead “←”) and white pulp area (denoted by arrowhead “→”), vacuolar degeneration (denoted by arrowhead “↑”). In the lung (**S-X**), the main lesions included bleeding (denoted by arrowhead “←”), alveolar septal rupture and thickening (denoted by arrowhead “→”), inflammatory cell infiltration (denoted by arrowhead “↓”). In the kidney (**Y-ZD**), the lesions mainly included hyperemia in the cortical area (denoted by arrowhead “←”) and medulla area (denoted by arrowhead “→”), shedding of renal tubular epithelial cells and glomerular swelling (denoted by arrowhead “↓”). In the brain (**ZE-ZJ**), the lesions mainly included cell edema (denoted by arrowhead “←”)

In heart, the myocardial fibers in the infected-untreated group were disordered and a large number of myocardial fibers were broken (Fig. 5B). The localized myocardial fiber arrangement was slightly disordered and broken in the acyclovir-treated group (Fig. 5C). The blood vessel wall of the heart was completely shed, and most of the myocardial fibers were broken in the KM-L group (Fig. 5D). In the KM-M (Fig. 5E) and KM-H (Fig. 5F) groups, varying degrees of disordered and broken myocardial fibers and localized vacuolar degeneration were observed. The uninfected-untreated group showed no obvious lesions (Fig. 5A).

In liver, PRV infection induced shedding blood vessel walls; liver cells were concentrated and swollen; liver cords were disorderly arranged, and the liver sinusoids became narrowed and diffused (Fig. 5H). The structure of hepatic cord was partly disappeared in the KM-L group (Fig. 5J). The KM-M (Fig. 5K), KM-H (Fig. 5L) and acyclovir (Fig. 5I) groups showed slightly localized separation of blood vessel wall, and the liver cells were slightly swollen and diffused. The uninfected-untreated group showed normal structure of liver (Fig. 5G).

In spleen, the red pulp area of the infected-untreated group was significantly reduced with vacuolar degeneration, and the white pulp area had abnormal and unclear boundaries (Fig. 5N). The red pulp area of the acyclovir group was decreased and the structure of the white pulp became unclear (Fig. 5O). The area of the red pulp in the KM-L group (Fig. 5P) was reduced, and the boundaries of the white pulp area were unclear. The area of white pulp in the KM-M group decreased, and slight vacuolar degeneration occurred (Fig. 5Q). The structure of the red pulp and white pulp in the KM-H group was normal, and only slight vacuolar degeneration occurred (Fig. 5R). The uninfected-untreated group showed normal structure of spleen (Fig. 5M).

In lung, PRV infection induced massive bleeding, inflammatory cell infiltration, and thickened alveolar septum in the infected-untreated group (Fig. 5T); After treatment with acyclovir and kaempferol, the lesions were alleviated (Fig. 5U and X); the uninfected-untreated group showed normal structure of lung (Fig. 5S).

In kidney, the cortical area is congested, and a large number of renal tubular epithelial cells are shedding and swollen in the infected-untreated group (Fig. 5Z). In the acyclovir group, the cortical area is mildly congested and the glomeruli are slightly swollen (Fig. 5ZA). Localized congestion and slightly shedding of renal tubular epithelial cells could be observed in the kaempferol-treated groups (Fig. 5ZB-ZD). The uninfected-untreated group showed normal structure of kidney (Fig. 5Y).

In brain, a small amount of cell edema occurred in each group (Fig.  5ZF-ZJ). The uninfected-untreated group showed normal structure of brain (Fig. 5ZE).

The pathological changes of each group were scored for tissue lesions. The results (Table 1) showed that compared with the infected-untreated group, kaempferol had different degrees of therapeutic effects on PRV-induced tissue lesions.

**Table 1:** Tissue lesions score on 5dpi

Group	Tissue lesions score					
	Heart	Liver	Spleen	Lung	Kidney	Brain
Infected-untreated control	6.34 ± 2.05	4.67 ± 0.94	6.33 ± 2.05	7.00 ± 1.41	6.33 ± 2.05	2.00 ± 0.87
KM-L	4.67 ± 0.94	3.67 ± 0.47	3.67 ± 0.47	3.67 ± 1.70	3.00 ± 0.82	1.33 ± 0.47
KM-M	4.34 ± 1.25	4.33 ± 1.25	4.67 ± 0.94	3.00 ± 0.82^*^	2.67 ± 0.94^*^	0.67 ± 0.94
KM-H	3.67 ± 0.47	3.00 ± 0.82	3.00 ± 0.82	2.67 ± 0.94^*^	2.33 ± 0.47^*^	1.33 ± 1.25
ACV	5.34 ± 0.94	4.33 ± 1.25	3.33 ± 0.47	3.33 ± 0.47^*^	3.67 ± 0.47	2.00 ± 1.41

Lesional scores of each organ (*n* = 4) were obtained by multiplying the degree of severity (0 = no lesions,1 = mild lesions, 2 = moderate lesions, and 3 = severe lesions) with the extent of lesions (1 = low extent, 2 = intermediate extent, and 3 = large extent)

*ACV* the acyclovir-treated group (160 mg/kg• body weight)

“*” represents the significant differences (*p* < 0.05) observed between the infected-untreated group and the treated groups

### The changes of serum cytokines

**Fig. 6:**
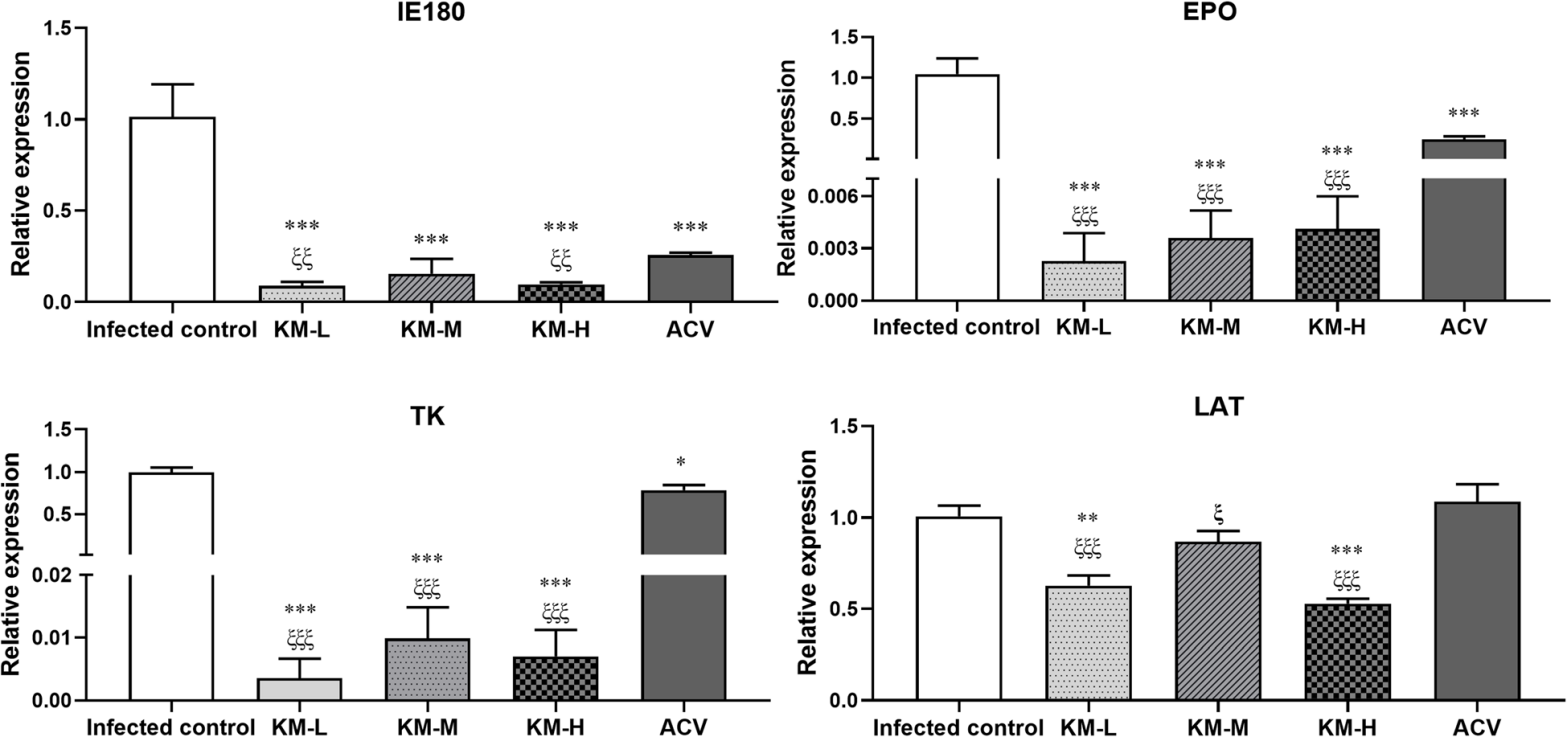
The concentrations of IL-1β, IL-6, TNF-α, IL-4 and IFN-γ in serum at 3, 4 and 5 dpi (*n* = 4). The concentrations of these cytokines were determined through ELISA. The KM-H, KM-M and KM-L represent the PRV-infected groups which were treated with kaempferol at doses of 240, 160 and 80 mg/kg• body weight, respectively. ACV, the acyclovir-treated group (160 mg/kg• body weight). Symbols “*, ** and ***” represent *p* < 0.05, *p* < 0.01 and *p* < 0.001, respectively, between the infected-untreated control and the infected-treated groups. Symbols “#, ## and ###” represent *p* < 0.05, *p* < 0.01 and *p* < 0.001, respectively, between the uninfected-untreated control and infected groups

Cytokines are involved in immune and inflammatory responses, which play a key role in protecting the body from foreign pathogens. TNF-α, IL-Iβ, IL-6 and IFN-γ, belonging to the pro-inflammatory cytokines, are involved in promoting acute inflammation to defense against infection [29]. PRV infection induced increased levels of TNF-α, IL-Iβ, IL-6 and IFN-γ. After treatment with kaempferol, the levels of IL-Iβ, IL-6 and IFN-γ (Fig. 6) were increased at 3 dpi in comparison with the uninfected-untreated and infected-untreated groups, and then gradually declined to normal levels at 4 and 5 dpi. The high dose of kaempferol group (240 mg/kg) still maintained higher levels of IL-Iβ and IFN-γ at 5 dpi. The TNF-α concentration showed a normal level in the treated groups except the high dose of kaempferol group (240 mg/kg) at 5 dpi. As one of the important anti-inflammatory factors, IL-4 can help fight viral infections. The IL-4 level was increased by PRV infection (Fig. 6). It was significantly increased by kaempferol-treatment in comparison with the uninfected-untreated and infected-untreated groups at 3 dpi, and then declined to the level of the infected-untreated group at 4 and 5 dpi (Fig. 6).

### Transcription levels of IE180, EPO, TK and LAT

**Fig. 7:**
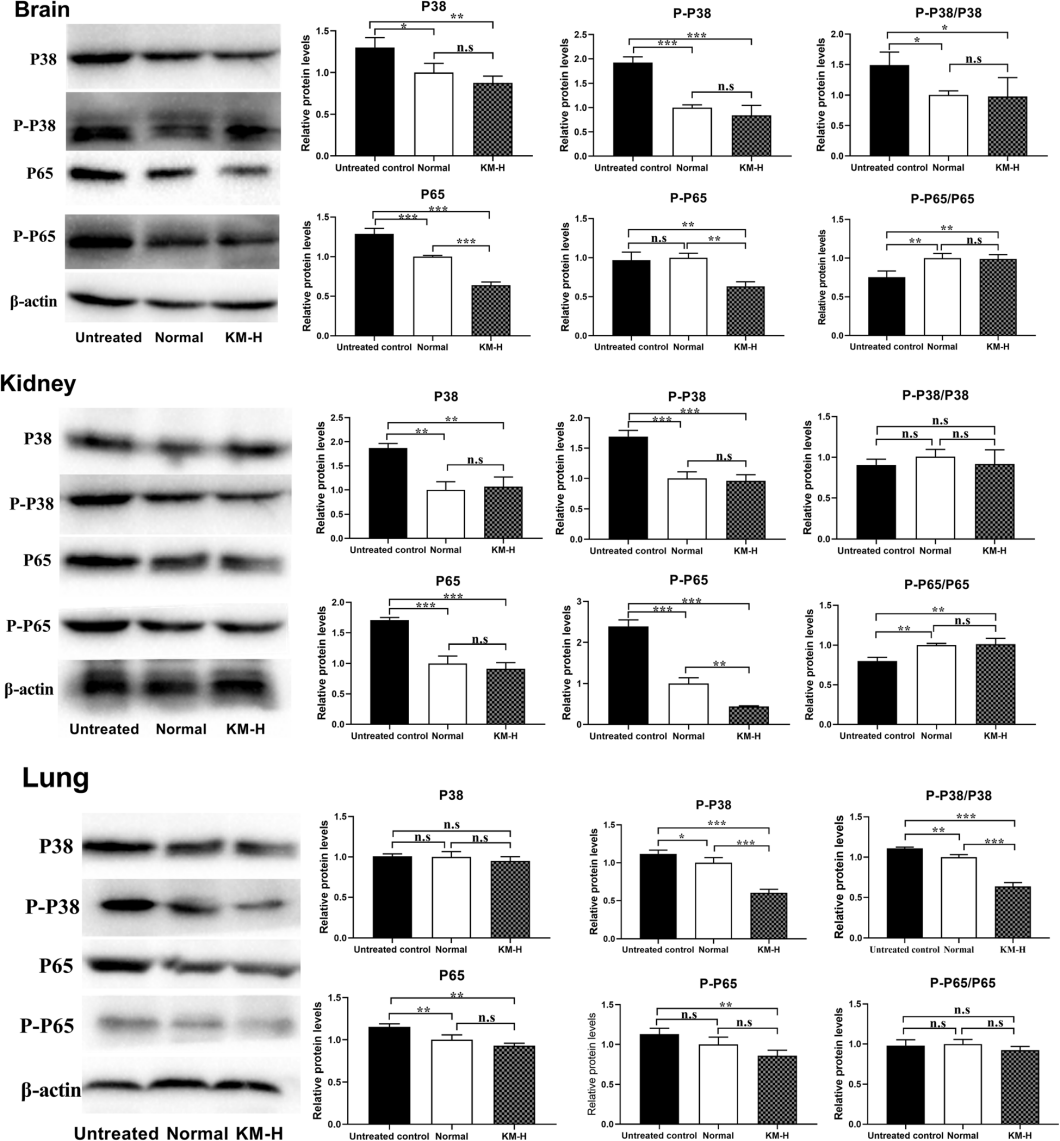
The gene expressions of IE180, EPO, TK and LAT in brain at 5 dpi (*n* = 4). Infected control, the infected-untreated control. The KM-H, KM-M and KM-L represent the PRV-infected groups which were treated with kaempferol at doses of 240, 160 and 80 mg/kg• body weight, respectively. ACV, the acyclovir-treated group (160 mg/kg• body weight). Symbols “** and ***” represent *p* < 0.01 and *p* < 0.001, respectively, between the infected-untreated control and the treated groups. Symbols “ξ, ξξ and ξξξ” represent *p* < 0.05, *p* < 0.01 and *p* < 0.001, respectively, between the acyclovir group and kaempferol groups

Viral gene transcription is the key step of virus replication; thus, it is an effective way to inhibit virus multiplication through blocking gene transcription. IE180 is the only immediate early gene of PRV and a transcriptional activator which can initiate the transcription of early genes [10, 30]. The transcription level of IE180 in the kaempferol and acyclovir groups were inhibited at 5dpi, leading to decreased expressions of the early genes (EPO and TK) (Fig. 7). LAT (latent related transcript), a large amount of RNA that is present and transcribed during the incubation period of PRV, plays an important role in the establishment, maintenance, and reactivation of PRV latency [31]. Compared with the infected-untreated group, kaempferol significantly reduced the expression of LAT, but acyclovir could not inhibit the LAT transcript (Fig. 7). Kaempferol showed higher inhibitory effects than acyclovir.

## Discussion

Since the 1980 s, the spread of AD worldwide had inevitably caused serious economic losses [32]. The farms in China currently rely on vaccination to prevent AD, including inactivated vaccines, attenuated vaccines and gene-deleted vaccines [17]. However, during the past decades, the PRV strain had mutated, and the existing vaccines were unable to control all infections, which eventually led to the repeated outbreak of AD. Recent case reports of human PRV infections suggested possible transmission to humans [4]. Therefore, development of alternative control measure is still the main task at present. Kaempferol is a very common dietary flavonoid compound that has inhibitory effects on several viruses, such as bovine herpesvirus 1 [33], HSV [34], Japanese encephalitis virus [35], and enterovirus 71 [36].

The elimination half time (t_1/2_) of kaempferol in total flavonoids of *Granati Pericarpium* in rat plasma was 10.124 h [37]. Another study showed that the t_1/2_ of kaempferol in rat serum was 8.3 h [38]. Therefore, in the present study, kaempferol was administered once a day in consideration of the t_1/2_. Kaempferol could effectively protect mice from PRV infection through improvement of survival rate by 22.22 % (Fig. 1). In addition to pigs, many mammals are susceptible to PRV, and often died after infection. Mice would die quickly after PRV infection, and the mortality rate is mostly 100 % within 3–7 dpi [39]. Previously, the antiviral activity of an herbal extract from *Kaempferia galanga* against PRV in mice were conducted and found that inoculation of 1 × 10^4^ TCID_50_ PRV could induce typical symptoms and the infected mice died within 7 days, which was in accord with the clinical feature of PRV-infected mice. The same result was observed in this study that the mice in the infected-untreated group had all died at 6 dpi. Acyclovir and its analogues are the most frequently used drugs approved for the treatment of HSV, which can inhibit the viral DNA polymerase [40]. Aciclovir also showed antiviral activity against other herpesviruses, such as varicella zoster virus, Epstein-Barr virus, cytomegalovirus and human herpesvirus 6 [41], thus it was served as positive control. In recent human cases, acyclovir was used for antiviral treatment of PRV-induced acute human encephalitis [4]. In this study, acyclovir exhibited antiviral activity against PRV in mice with survival rate of 16.67 %, but it is lower than kaempferol which suggested a potential application of kaempferol in control of PRV infections in animals and humans.

There are different routes used for inoculating mice with PRV, which could lead to different clinical symptoms and pathological changes. Intraperitoneal injection, scarification and intranasal drip are commonly used routes. Infections through nasal dripping or intracranial inoculation can spread quickly in the brain and lead to neurotropic lesions, which could be used to build a viral encephalitis model [42, 43]. Inoculation with PRV by flank scarification in mice could induce frantic scratching and biting of the skin at the inoculation site, and little infectious virions or viral antigens were detected in the brain [27]. By intraperitoneal injection of PRV, the virus would proliferate from the injection site and spread to organs and brain with obvious clinical symptoms of scratching and biting the injection site [44]. The purpose of this study was to explore how kaempferol inhibited PRV proliferation in organs, thus the intraperitoneal injection was performed. In addition, other inoculation routes for PRV were also used in mice, such as plantar inoculation [45] and intramuscular injection [46].

Viral load is a direct parameter in the evaluation of antiviral effects *in vivo*, which can reflect virus replication in different organs [47–50]. In this study, PRV reproduction in the test organs, including brain, heart, spleen, lung and kidney, were evaluated. The results (Fig. 3) showed that the virus titer in brain was the highest, which was about 50-fold than other test organs. The reason could be attributed to PRV belonging to a neurotropic alphaherpesvirus which could invade the CNS via trigeminal nerve, as well as by sympathetic and parasympathetic pathways [51]. The main feature of PRV infection is encephalomyelitis, which is often accompanied by inflammation of the upper respiratory tract and lungs [52]. Kaempferol could significantly inhibit virus replication in the test organs, especially in the brain that the viral gene copies were decreased by more than 700-fold. In contrast, acyclovir showed lower ability to inhibit virus reproduction than kaempferol, and the viral gene copies were decreased by about 10-fold in the brain. The immunohistochemical study also demonstrated that the amount of progeny virus in brain was markedly decreased after kaempferol or acyclovir treatment (Fig. 4). In the antiviral effects of resveratrol in piglets infected with PRV, resveratrol could significantly inhibit PRV replication in brain, and the levels of viral copies were positively linked to the clinical parameters of infected piglets [53]. In this study, kaempferol was effective to delay and inhibit the clinical symptoms in the PRV-infected mice which may attributed to inhibition of virus replication in brain. IHC is an intuitive way to evaluate the distribution of virus replication in brain cells. The results showed that both acyclovir and kaempferol treatment could significantly reduce the proportion of positive cells in brain. The trend that PRV replication was significantly inhibited *in vivo* was not changed in the whole text. Indeed, there were some inconsistencies in the four treated groups (KM-H, KM-M, KM-L and acyclovir), such as the viral load and survival rate. This should be attribute to the complicated effects of the test drugs (Acyclovir and kaempferol) *in vivo*, and animal individual differences were also one of the mayor factors leading to this inconsistency. In the present study, the effects of kaempferol on PRV infection was confirmed, and recommended dosage was obtained for further research.

After mice were infected with PRV, histopathological analysis found that the brain, spleen and liver tissues had different degrees of lesions [54]. Studies have shown that PRV could cause lethal respiratory disease in an animal model of PRV-infected BALB/c mice [55]. In the present study, mild tissue damages were observed in the PRV-infected mice after treated with kaempferol (Fig. 5), indicating that kaempferol could alleviate the histopathological changes.

Although an inflammatory response is the first line of defense to prevent the spread of viral infections, the main challenge is to ensure that inflammation is resolved [38], then the body’s homeostasis can be restored to normal. When the inflammatory response is uncontrolled, it usually leads to more severe inflammation, which may cause damages to the host [56]. In this study, the levels of pro-inflammatory cytokines, including IL-1β, IL-6, TNF-α and IFN-γ, were elevated after infection, suggesting the innate immunity was activated by PRV. After kaempferol treatment, the levels of these pro-inflammatory cytokines were higher than the infected control at 3 dpi, and then almost declined to the normal level at 5 dpi. This suggested that kaempferol could enhance the inflammatory response at the early stage of infection to inhibit viral replication, then recovered it to normal to avoid severe inflammation. The anti-inflammatory cytokine IL-4, produced by Th2 cells, NKT cells, basophils and mast cells, has a wide range of immunological functions, such as regulating the function of macrophage [57, 58]. Studies have shown that IL-4 could suppress PMA-induced HIV expression at the transcriptional level in monocytic U1 cell [59]. PRV infection increased the IL-4 level in mice. After kaempferol treatment, IL-4 level was significantly higher than that of the infected-untreated control at 3 dpi, indicating that kaempferol could regulate the IL-4 level in PRV-infected mice.

After PRV enters into the host cells, the capsid is transported along microtubules to the cell nucleus and the viral DNA is injected into the nucleus [52]. Then, the only immediate-early gene of PRV, IE180, is transcribed directly following infection [10]. The product of IE180 gene was the transactivator required for transcription of early genes [10, 30]. In this study, the transcriptional levels of early genes, EPO and TK, were inhibited by kaempferol in the brain of PRV-infected mice, which may indicate that the function of IE180 protein was blocked (Fig. 7). After the body is infected with the virus for the first time, the virus can establish latent infection in the sensory ganglion and brain [60]. With the activation of latent infection, a continuous inflammatory response could be triggered, which can directly cause damage to the nerve tissue [61]. LAT plays an important role in the establishment, maintenance, and reactivation of PRV latency. It inhibits the expression of the immediate early gene IE180 during incubation period, which could prevent the virus from entering the lytic infection period [62, 63]. The present study found that the transcriptional level of LAT in brain was inhibited, suggesting that kaempferol could inhibit PRV latency in mice. Currently, there is no drug available which could allow eradication of the virus from the infected body. Approved therapies for HSV-1 infection such as acyclovir are the inhibitors of the viral DNA replication. Although they are effective against primary infection, they cannot protect against the virus in the latent state, resulting in the reoccurrence of the disease [64]. That could be the reason why acyclovir group had high transcriptional level of LAT.

## Conclusions

Kaempferol exhibits potent antiviral activity against PRV in mice, which is better than acyclovir. Kaempferol can increase the survival rate, reduce virus titer and gene expressions, and alleviate tissue lesions in PRV-infected mice. The anti-PRV activity attributes to regulation of immune response. Kaempferol exhibits the potential to control PRV infection.

## Methods

### Virus and chemicals

PRV (Ra strain) is a classical PRV strain from the China Veterinary Culture Collection Center (Beijing, China). It was propagated for five generations in PK-15 cells and preserved in the Natural Medicine Research Center Sichuan Agricultural University (Chengdu, China) [53], and the 50 % tissue culture infective dose (TCID_50_) was determined as 10^− 7.43^ / mL. The virus was diluted to 1 × 10^4^ TCID_50_ with PBS before use. Kaempferol (No. MB2171) and acyclovir (No. MB1002) were purchased from Dalian Meilun biotechnology Co., Ltd. (Dalian, China) and dissolved in 0.5 % carboxy methyl cellulose sodium solution.

### Animals and experimental design

 The experimental protocol was approved by the National Institute of Animal Health Animal Care and Use Committee at Sichuan Agricultural University (approval number 2018-012).

One hundred eighty male specific pathogen-free KM mice (body weight 20 ± 2 g) were commercially obtained from the Chengdu Dossy Experimental Animals Co., Ltd. (Chengdu, China), and kept in the BSL-2 lab at Sichuan Agricultural University (Ya’an, China). They were housed at 20–25^°^C with a relative humidity of 55 ± 5 % and a 12 h light-dark cycle. After acclimating for a week, the mice were randomly divided into the following 6 groups (*n* = 30): low dose of kaempferol group (KM-L, 80 mg/kg), medium dose of kaempferol group (KM-M, 160 mg/kg), high dose of kaempferol group (KM-H, 240 mg/kg), acyclovir group (ACV, 160 mg/kg), infected-untreated group and uninfected-untreated group. All mice, except those in the uninfected-untreated group (0.1mL PBS), were intraperitoneally injected with 0.1mL of 1 × 10^4^ TCID_50_ PRV. The treatment was started at 1 h post infection. The mice in the treated groups were orally administered with 0.2 mL kaempferol or Acyclovir once a day for 6 successive days. In the infected-untreated group and uninfected-untreated group, the mice received 0.2mL 0.5 % CMC-Na. Four mice were randomly selected in each group for sample collection at 3, 4 and 5 days post-infection (dpi), respectively. Blood sample collection was performed by retro-orbital puncture under anaesthesia by isoflurane inhalation. In order to minimize animals suffering during blood samples collection, an eye drop of tetracaine 1 % was applied. Then, animals were euthanized by cervical dislocation and subjected to full dissection.

### Survival rate

The number of deaths in each group was recorded daily. The survival rate was calculated as follows:

$$ < mathdollar>\mathrm{Survival}\kern0.34em \mathrm{rate}=\mathrm{number}\kern0.34em \mathrm{of}\kern0.34em \mathrm{surviving}\kern0.34em \mathrm{mice}/\mathrm{total}\kern0.34em \mathrm{number}\kern0.34em \mathrm{of}\kern0.34em \mathrm{mice} $$

### Organ coefficient

After dissection, the heart, liver, spleen, lung, kidney, thymus and brain were exercised and weighed. The relative organ weight was calculated according to the formula:

$$\mathrm{Organcoefficient}(\mathrm{mg}/\mathrm g)=\mathrm{organweight}/\mathrm{bodyweight}.$$

### Viral load assay

The liver, heart, spleen, brain, lung and kidney were collected from each group and immediately frozen with liquid nitrogen, followed by homogenization. Total DNA of tissue sample (25 mg) was extracted by DNA Extraction Kit (Biomed DL107-01; Beijing, China) according to the manufacturer’s instructions. The viral copies were determined by the fluorescent quantitative polymerase chain reaction (FQ-PCR) method described by Zhao et al., [65]. The FQ-PCR was performed at 95 °C for 120 s, 95 °C for 5 s and 56.5 °C for 30 s (40 cycles) by using a CFX connect™ real-time PCR detection system (Bio-Rad, USA).

### Serum cytokines assay

Blood samples collected from eyeball were allowed to coagulation at room temperature for 30 min. Serum was separated by centrifugation at 3000×ɡ for 5 min. The levels of IL-1β, IL-4, IL-6, TNF-α and IFN-γ in serum were measured using the ELISA kits according to the manufacturer’s instructions (Beijing Gersion Bio-Technology Co., Ltd, China).

### Histopathological examination

During dissection, the heart, liver, spleen, lung, kidney and brain were taken and fixed in 4.0 % paraformaldehyde for 2 days, followed by embedding in paraffin. Section (4 μm) were cut and stained with hematoxylin-eosin (HE) solution. Histopathological changes were observed under a microscope (Nikon eclipse 80i, Tokyo, Japan). Three slides containing sections randomly selected from different part of each sample, with an area of 2000–2500 mm^2^ per slide, were analyzed to determine the lesion scores of each organ. The whole lesions for each tissue were scored by multiplying the degree of severity (0 = no lesions, 1 = mild lesions, 2 = moderate lesions, and 3 = severe lesions) with the extent of lesions (1 = low extent, 2 = intermediate extent, and 3 = large extent) [66]. For each organ, three slides from different part of each tissue were analyzed, the maximal score was 9 and the minimal score was 0.

### Immunohistochemistry

The tissue slides of brain from different groups were deparaffinised in xylene, followed by dehydration with ethanol. Endogenous peroxidase activity was blocked by treatment with 0.3 % H_2_O_2_ in methanol for 20 min at RT. After antigen retrieval, the tissue slides were blocked with 5 % goat serum for 20 min at RT and then probed with anti-Pseudorabies Virus antibody (No. ab3534; Abcam, England) at 4℃ overnight. After washing, the tissue slides were incubated with biotinylated goat anti-rabbit IgG (No. ab205718; Abcam, England) at RT for 1 h, then stained with DAB and counterstained with haematoxylin. Finally, the slides were evaluated under a microscope (Nikon, Japan).

### Gene expression assay

The expressions of PRV genes related to transcription and latent infection (IE180, EPO, LAT and TK) were detected through real-time PCR assay. Briefly, total RNA from brain was extracted using the RNAiso Plus (No.9108; TaKaRa, China) according to the manufacturer’s instructions. Equal amounts of the RNA samples were immediately reverse transcribed into cDNA using the RevertAid First Strand cDNA kit (No. K1622; Thermo Scientific™) according to the manufacturer’s instructions. The primers were list in Table 2. The real-time PCR was performed with a CFX connect™ real-time PCR detection system (Bio-Rad, USA) using the iQ SYBR Green Supermix kit (Bio-Bad, USA) at 95 °C for 45 s, followed by 39 cycles of 95 °C for 7 s and 62 °C (60 °C for TK gene) for 34 s. A melting curve of the products (55–95 °C) was also conducted to confirm the absence of artefacts. The relative expression levels of the target genes were calculated by the 2^−ΔΔCT^ method using Bio-Rad CFX Manager software.

**Table 2:** The primers used for real-time PCR

Gene name	Primer sequence (5’→3’)	T_m_ (℃)
β-actin	F: GGCTGTATTCCCCTCCATCG R: CCAGTTGGTAACAATGCCATGT	62
EPO	F: GGGTGTGAACTATATCGACACGTC R: TCAGAGTCAGAGTGTGCCTCG	62
IE180	F: CATCGTGCTGGACACCATCGAG R: ACGTAGACGTGGTAGTCCCCCA	62
LAT	F: GGCAGCAGGACTACTGTCA R: GTCTTGGTGGGAAGAAGTA	62
TK	F: ATGACGGTCGTCTTTGACCGCCAC R: CGCTGATGTCCCCGACGATGAA	60

*F* forward primer, *R* reverse primer

### Statistical analysis

Results were expressed as means ± standard deviation (SD). Significant differences were determined using a one-way analysis of variance (ANOVA) followed by Duncan’s Multiple Range test in SPSS 19.0 (IBM Corp., Armonk, NY, USA) at *p* < 0.05 for significance.

## Data Availability

The datasets used and/or analyzed during the current study are available from the corresponding author upon reasonable request.

## Abbreviations

PRV: Pseudorabies virus
AD: Aujeszky’s disease
LAT: Latency-associated transcript
TCID_50_: 50 % tissue culture infective dose
ACV: Acyclovir
CMC-Na: Carboxymethylcellulose sodium
HE: Hematoxylin-eosin
KM-L: Low dose of kaempferol group
KM-M: Medium dose of kaempferol group
KM-H: High dose of kaempferol group
ELISA: Enzyme-linked immunosorbent assay
